# Proprotein Convertase Subtilisin Kexin 9 Inhibitor in Severe Sepsis and Septic Shock Patients in a Phase II Prospective Cohort Study—Preliminary Results

**DOI:** 10.3390/idr16060083

**Published:** 2024-10-24

**Authors:** Ziv Rosman, Yasmin Maor, Iris Zohar, Gingy Ronen Balmor, Miri Schamroth Pravda, Adam Lee Goldstein, Milena Tocut, Arie Soroksky

**Affiliations:** 1 Intensive Care Department, E. Wolfson Medical Center, Holon 5822012, Israel; gingyb@wmc.gov.il (G.R.B.); mirisc@wmc.gov.il (M.S.P.); aries@wmc.gov.il (A.S.); 2 Faculty of Medicine, Tel Aviv University, Tel Aviv 6997801, Israel; yasminm@wmc.gov.il (Y.M.); irisz@wmc.gov.il (I.Z.); milenat@wmc.gov.il (M.T.); 3 Infectious Disease Unit, E. Wolfson Medical Center, Holon 5822012, Israel; 4 Surgical Department A Trauma Division, E. Wolfson Medical Center, Holon 5822012, Israel; adamleeg@wmc.gov.il; 5 Internal Medicine C Department, E. Wolfson Medical Center, Holon 5822012, Israel

**Keywords:** PCSK9 inhibitor, sepsis, septic shock, alirocumab

## Abstract

Sepsis is a life-threatening organ dysfunction syndrome caused by a dysregulated host response to infection that has a high mortality rate. Proprotein convertase subtilisin kexin 9 (PCSK9) is a serine protease secreted by the liver. Its binding to the low-density lipoprotein (LDL) receptor enhances its degradation, causing an increase in LDL levels in the blood. **Objectives**: Administering a PCSK9 inhibitor leading to an increase in lipid uptake by the liver may positively affect septic patients due to the increased removal of endotoxins. **Methods**: This preliminary study aimed to examine the safety of PCSK9 inhibitor use in septic and septic shock patients. We treated five septic patients in the intensive care unit with 300 mg of alirocumab following serious adverse events for 28 days. **Results**: Four of our patients did not experience any adverse events, and all of them survived. One patient died after discharge from the intensive care unit, and this death was presumably not related to the study drug. The patients rapidly recovered from the inflammatory stage of sepsis. **Conclusions**: Alirocumab appears safe in severe sepsis and septic shock patients. The outcome data are promising. Only a basic safety profile can be assessed based on this pilot study. Further study with a PCSK-9 inhibitor in septic or septic shock patients is required to further determine its benefit in ICU patients.

## 1. Introduction

Sepsis is a life-threatening organ dysfunction syndrome caused by a dysregulated host response to infection that has a high mortality rate [1]. The role of bacterial endotoxin is known to be central to the development of septic shock in Gram-negative bacterial sepsis [2]. The membrane of Gram-negative bacteria is made of lipopolysaccharides (LPS), which serve as an endotoxin. The pattern recognition receptor for LPS is Toll-like receptor 4 (TLR4) [3,4], which initiates the inflammatory cascade upon activation.

In past studies, despite the lack of LPS on Gram-positive bacteria, mice with mutant TLR4 had a greater bacterial burden and lower survival rates, suggesting a role for TLR4 in the inflammatory cascade despite the lack of LPS [5].

This may occur through the direct binding of superantigens to the MHC-II receptor and T cell receptor, causing massive T cell activation and bypassing antigen-presenting cells, leading to a cytokine storm [4]. Murine models assessing cytokine response during sepsis and inflammation have demonstrated high levels of low-density lipoprotein (LDL) in the blood due to the suppression of LDL receptor proteins in the liver [6].

Proprotein convertase subtilisin kexin 9 (PCSK9) is a serine protease secreted by the liver. Its binding to the LDL receptor enhances its degradation, causing an increase in LDL levels in the blood [7,8]. In the absence of PCSK9, the number of LDL receptors on the liver cell surface increases, and more circulating LDL is removed from the plasma [9]. PCSK9 expression increases during inflammation [10]. In addition to its effects on LDL metabolism, various studies have revealed additional roles of PCSK9 in different stages of atherosclerosis, including its ability to target other members of the LDLR family. PCSK9 from plasma and vascular cells can contribute to the formation of atherosclerotic plaques and thrombosis by promoting platelet activation, leukocyte recruitment, and clot formation through mechanisms unrelated to systemic lipid changes [9,10].

Sepsis is a critical condition resulting from an exaggerated immune response to infection that causes multiple organ dysfunction, including in regions far from the original infection site. Pathogen-derived lipids entering the bloodstream can initiate systemic inflammatory response syndrome (SIRS). Clinical research has highlighted PCSK9 as a critical player in the pathogenesis of sepsis, with findings indicating that inhibiting PCSK9 activity can substantially enhance sepsis outcomes. Consequently, PCSK9 could serve as a promising biomarker for assessing a sepsis prognosis. For instance, elevated serum PCSK9 levels in sepsis patients have been closely linked to the development of multiple organ failure [11,12].

Microbial pathogenic lipids, namely, LPS in Gram-negative bacteria, lipoteichoic acid in Gram-positive bacteria, and phospholipomannan in fungi, are bound to lipids in the blood, causing an increase in PCSK9 in plasma. This has led to the speculation that increased lipid clearance by the liver leads to increased LPS clearance, affecting the process of sepsis and septic shock [11,12,13]. Thus, administering a PCSK9 inhibitor, leading to an increase in lipid uptake by the liver, may positively affect septic patients.

The benefit of inhibiting PCSK9 in sepsis was further strengthened by a study by Walley et al. [14] examining septic patients who had at least one PCSK9 loss-of-function allele. The survival rate of these patients increased over a 28-day period compared to that of patients with gain-of-function alleles [14].

Most of the information regarding PCSK9 inhibitors comes from studies assessing their ability to lower LDL levels in patients at high risk for cardiovascular events. In these studies, which included tens of thousands of patients, the drug had a good safety profile. The ODYSSEY LONG TERM study [15], which included 2341 patients and examined the safety of the PCSK9 inhibitor alirocumab at a dose of 150 mg, demonstrated similar rates of adverse events between the treatment group and the placebo group. In a recent large meta-analysis examining the adverse effects of PCSK9 inhibitors, no statistically significant events regarding neurocognitive function or diabetes were observed [16]. Similar results were demonstrated in a small postmarketing study comparing a placebo, 75 mg of alirocumab, and 150 mg of alirocumab [17]. Additional evidence for the safety and clinical benefit of alirocumab in treating sepsis was noted in a study performed with COVID-19 patients [18]. We sought to determine the safety of administering a subcutaneous PCSK9 inhibitor (alirocumab) at a single dose of 300 mg in patients with severe sepsis or septic shock.

## 2. Materials and Methods

The trial is an investigator-initiated open-label phase II study examining the safety of administering 300 mg of a subcutaneous (SC) PCSK9 inhibitor (alirocumab) once in septic and septic shock patients upon admission to the intensive care unit (ICU) during a two-month period (June–July 2023).

This was the first stage of this study prior to expanding it to a randomized controlled study with a larger sample size testing the hypothesis that administering a PCSK9 inhibitor within 48 h of fulfilling the inclusion criteria improved patient outcomes.

This study was approved by the Helsinki Ethical Committee at Wolfson Medical Center, (study no. 101639). A waiver for informed consent was received due to the state of the patients, and their families were informed of the recruitment.

Patients diagnosed with sepsis or septic shock according to the latest sepsis guidelines [19] were screened upon admission to the ICU. Patients who met the following criteria were included: aged 18 to 85 years, had sepsis or septic shock, and had at least one organ failure. 

We excluded patients with known hypersensitivity to alirocumab, patients with liver injury (defined as Child–Pugh score class C) due to the reported potential hepatotoxic side effects of PCSK9, patients with an estimated glomerular filtration rate (GFR) of less than 20 mL/min/1.73 m, patients with any terminal illness with a life expectancy of fewer than 6 months, breastfeeding women or pregnant women (this was a safety preliminary study, and we did not recruit pregnant patients), and patients who were expected to die within 24 h of ICU admission.

After recruitment, 300 mg of subcutaneous alirocumab was administered within 48 h of the patients fulfilling the inclusion criteria. Follow-up continued until discharge from the hospital, or up to 28 days.

In addition to the study drug, patients received standard treatment, including antibiotics, according to common practice guidelines.

We recorded the clinical severity scores of the SOFA and APACHE II scores. Clinical and laboratory data were collected prospectively from patients’ files. Any serious adverse events were recorded and reported.

Outcomes: The primary outcome was the safety profile of alirocumab in patients with sepsis or septic shock, which was measured as the rate of serious adverse events.

The secondary outcome was the length of ICU stay, duration of mechanical ventilation, 28-day ICU mortality, total hospital mortality, rate of nosocomial infections, SOFA score difference from day 0 (treatment day) until day 7, difference in blood lactate levels from day 0 (treatment day) until day 3, and the following markers of inflammation: interleukin 6 (IL6), C reactive protein (CRP), procalcitonin (PCT), and WBC.

## 3. Results

This study was conducted over two months. Nine patients were screened, of which five patients were recruited for this study.

**Figure 1:**
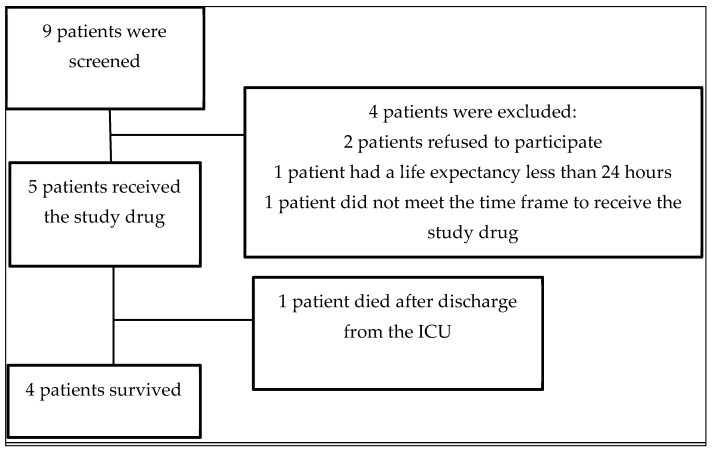
Patient recruitment.

Two patients were excluded due to the refusal of the patient’s family, one due to a life expectancy of less than 24 h, and one because she did not meet the time frame for the administration of the drug (Figure 1).

The patients’ characteristics on admission to the ICU are presented in Table 1. The patients’ mean age was 56.2 (SD 10.7). The patients’ respiratory involvement was variable in severity. The fifth patient required a tracheostomy due to his medical condition at the time of admission. Since the study drug was administered subcutaneously (SC), we recruited patients with sepsis or septic shock who required mild to moderate doses of noradrenaline. We wanted to limit the possibility of impaired drug absorption from SC tissue due to severe vasoconstriction due to severe septic shock. The noradrenaline doses ranged between 10 mcg/min and 50 mcg/min.

All patients survived to ICU discharge. Patient number one had an unexpected death 23 days after drug administration (4 days after being discharged from the ICU). Upon discharge, the patient’s inflammatory markers were normal, with a CRP of 4.1 mg/L. This patient completely recovered from his infection and was about to be discharged from the hospital.

Despite severe septic shock and mechanical ventilation, none of the patients developed acute respiratory distress syndrome (ARDS).

No adverse events were recorded, except for patient number one, as reported, with a sudden unexplained death. Despite his unexplained death, it seems that it was not related to the infectious condition or the study drug.

All patients except patient 2 decreased their inflammatory markers, as can be seen from the levels of IL-6, CRP, and LDL.

**Figure 2:**
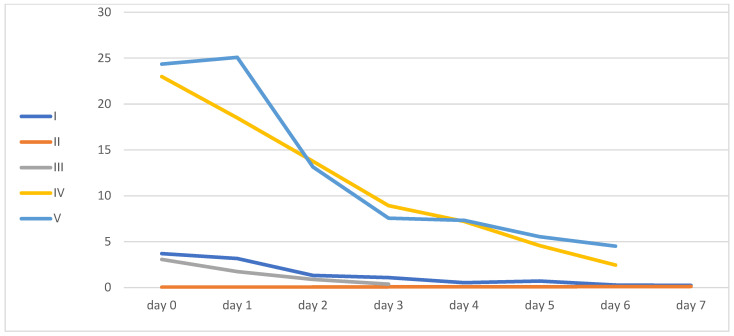
Procalcitonin levels.

**Figure 3:**
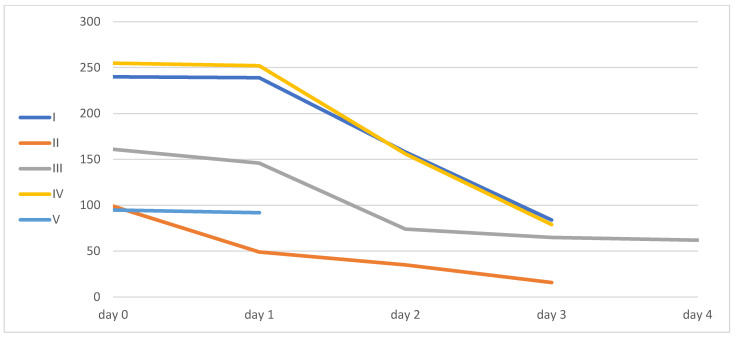
IL-6 levels. Patient V’s IL-6 samples were partially lost.

**Figure 4:**
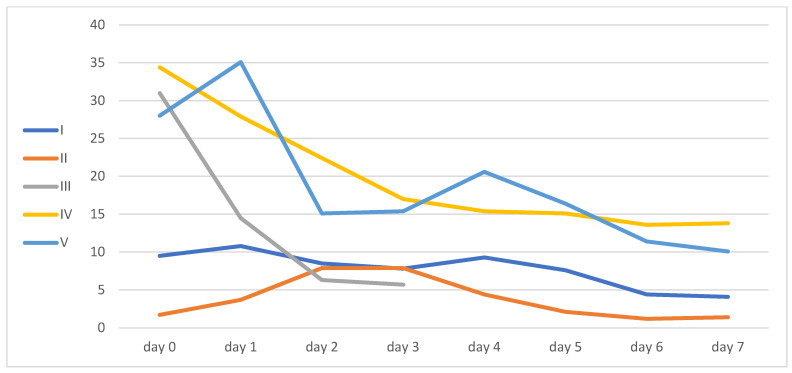
CRP levels.

**Figure 5:**
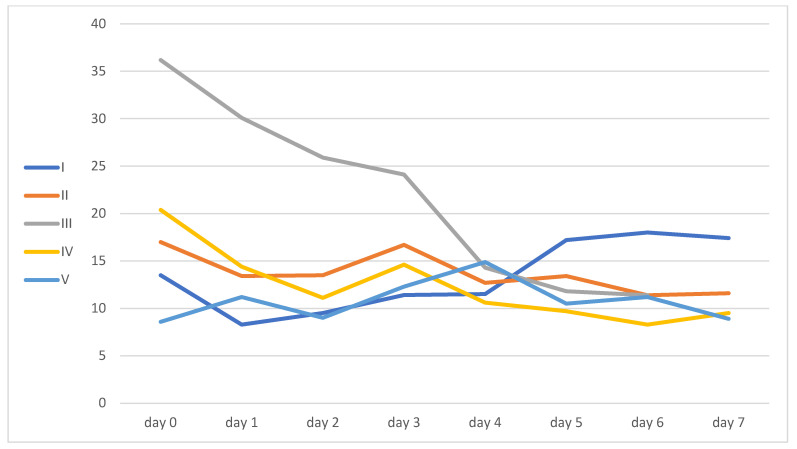
WBC levels.

Only patient number four had a kidney injury, and it improved during his ICU hospitalization. Inflammatory markers and liver function enzyme levels are shown in Table 2 and Figure 2, Figure 3, Figure 4 and Figure 5.

## 4. Discussion

In this preliminary phase 2 study assessing the safety of the PSCK9 inhibitor alirocumab in patients with severe sepsis and septic shock, five patients were included; none of these patients experienced severe adverse events even remotely related to the administered drug, and all survived their stay in the ICU, despite having a variety of severe infectious diseases, including infections, with a high mortality rate. One patient (patient number one) had a sudden unexplained death 23 days after drug administration during a period in which the drug effect was minimal, as the half-life of the drug is up to 20 days [20]. This patient completely recovered from his infectious disease and was a candidate for hospital discharge. We assumed that his unexpected sudden death was related to recreational drug use, as he was a known intravenous drug user.

In other studies assessing alirocumab, the rate of sudden death was zero and did not differ from that in the placebo arms [17,18]. Thus, we assume that this unfortunate death was not related to alirocumab administration.

We observed a rapid decrease in inflammatory markers, along with a relatively rapid decrease in the required vasopressor dose. This coincided with a relatively short duration of ventilatory support. Furthermore, none of the patients developed ARDS. In addition, one patient developed a nosocomial infection, ventilation-acquired pneumonia that developed 9 days after drug administration, after prolonged mechanical ventilation, and the patient recovered within 28 days of follow-up. No local or systemic allergic reactions were observed.

All patients had two organ failures, except for the third patient, who had the shortest stay in the ICU and the mildest disease. This allowed us to examine the effect of the drug on patients with a variety of disease severities. The second patient did not have high initial inflammatory marker levels and had mild disease. The use of PCSK-9 inhibitors did not have any adverse effects on kidney function, as seen in patient four, who improved his kidney function despite the treatment.

Further support for the safety and benefit of PCSK-9 in patients with severe COVID-19 was demonstrated in a recent study [18]. In this double-blind randomized controlled study, patients who were diagnosed with COVID-19 and pneumonia and treated with 300 mg of alirocumab were included. The PCSK-9 inhibitor decreased the inflammatory response, as shown by the decreased levels of IL-6 in the presence of the study drug, and decreased the mortality rate by as much as half the mortality in the study drug group. No adverse events or serious adverse events were reported, as in our study.

### Limitations

Our study has several limitations, including a very small size. It is possible that additional safety issues will arise when the sample size increases. We deliberately conducted a pilot study for the maximal safety of the patients. Furthermore, this study was not blinded and had no control, which eliminated the option for any conclusions to be made from our findings besides, perhaps, a trend. Aside from that, an obvious selection bias cannot be eliminated in such a study.

As patients were sedated, some of the mild adverse events of alirocumab could not be assessed, but these side effects are not clinically significant for ICU patients. Also, this study is too small to indicate the clinically beneficial effect of alirocumab, and further study is needed for this outcome.

## 5. Conclusions

Alirocumab appears to be safe in severe sepsis and septic shock patients. The outcome data are promising. Only a basic safety profile can be assessed based on this pilot study.

Further study with a PCSK-9 inhibitor in septic or septic shock patients is required to further determine its benefit in ICU patients. A larger multicenter blinded randomized control study is underway.

## Figures and Tables

**Table 1:** Patients’ baseline characteristics.

	Patient I	Patient II	Patient III	Patient IV	Patient V
Age—years	44	59	58	74	46
Sex	Male	Male	Male	Male	Male
Body mass index	32.7	32	19	28	31
Diagnosis on admission	Deep tissue infection	Pneumonia	Pneumonia	Infected skin wound	Deep tissue infection
Infectious agent	Streptococcus Pyogenes Group A	Streptococcus Pneumoniae	Staphylococcus Aureus	Klebsiella Pneumoniae	Streptococcus Group F
Admission type	Surgical	Medical	Medical	Medical	Surgical
Medical history	Intravenous drug user	COPD	COPD, CHF	Chronic kidney disease	none
APACHE II on admission	20	21	12	8	4
SOFA on admission	9	9	2	5	4
Days until fulfilling inclusion criteria from hospital admission	3	1	1	2	0
Organ failure on recruitment	2	2	1	2	2
Mechanical ventilation upon recruitment	yes	yes	no	No	yes
P/F Ratio	345	90	111	236	270
Vasopressors at recruitment	Yes	yes	no	yes	Yes
Fluid balance at recruitment	279 mL	442 mL	1960 mL	4909 mL	1750 mL

**Table 2:** Patients’ laboratory results.

	Patient I	Patient II	Patient III	Patient IV	Patient V
IL-6 on recruitment (pg/mL)	255	65	240	49	95
IL-6 on day 5	79	146	84	16	92 *
CRP on recruitment (mg/L)	9.5	1.7	31	34.4	28
CRP on day 7	4.1	1.4	missing data	13.8	16.1
PCT on recruitment (ng/mL)	3.72	0.05	3.08	23	24.36
PCT on day 7	0.24	0.31	missing data	2.47	4.53
WBC on recruitment (K/microL)	13.5	17	30.1	20.4	8.6
WBC on discharge	17.4	11.6	11.4	9.4	14.1
LDH on recruitment (iu/L)	976	349	353	543	849
LDH on discharge	646	373	425	767	305
AST on recruitment (iu/L)	84	16	20	43	94
AST on day 7	48	23	21	44	52
ALT on recruitment (iu/L)	85	36	10	29	62
ALT on day 7	42	53	16	37	26
Creatinine on recruitment (mg/dL)	0.65	0.7	0.38	4.66	0.94
Creatinine on day 7	2.02	0.63	0.38	3.48	0.58

* Patient V’s IL-6 levels were taken only on recruitment and on day 2. Some data are missing due to a lack of adherence to the study protocol.

## Data Availability

The datasets used and/or analyzed during the current study are available from the corresponding author upon reasonable request.

## References

[1] Singer M., Deutschman C.S. (2016). The Third International Consensus Definitions for Sepsis and Septic Shock (Sepsis-3). JAMA.

[2] Sriskandan S., Cohen J. (1999). Gram-positive sepsis: Mechanisms and Differences from Gram-Negative Sepsis. Infect. Dis. Clin. North Am..

[3] Medzhitov R., Preston-Hurlburt P. (1997). A human homologue of the Drosophila Toll protein signals activation of adaptive immunity. Nature.

[4] Ramachandran G. (2014). Gram-positive and gram-negative bacterial toxins in sepsis. Virulence.

[5] Branger J., Knapp S. (2004). Role of Toll-Like Receptor 4 in Gram-Positive and Gram-Negative Pneumonia in Mice. Infect. Immun..

[6] Liao W., Rudling M. (1996). Endotoxin suppresses rat hepatic low-density lipoprotein receptor expression. Biochem. J..

[7] Horton J.D., Cohen J.C. (2007). Molecular biology of PCSK9: Its role in LDL metabolism. Trends Biochem. Sci..

[8] Seidah N.G., Awan Z., Chréti M., Mbikay M. (2014). PCSK9: A key modulator of cardiovascular health. Circ. Res..

[9] Gouni-Berthold I., Descamps O.S. (2016). Systematic review of published Phase 3 data on anti-PCSK9 monoclonal antibodies in patients with hypercholesterolaemia. Br. J. Clin. Pharmacol..

[10] Feingold K.R., Moser A.H. (2008). Inflammation Stimulates the Expression of PCSK9. Biochem. Biophys. Res. Commun..

[11] Dos Santos C., Marshall J.C. (2014). Bridging lipid metabolism and innate host defense. Sci. Transl. Med..

[12] Norata G.D., Tavori H. (2016). Biology of proprotein convertase subtilisin kexin 9: Beyond low-density lipoprotein cholesterol lowering. Cardiovasc. Res..

[13] Walley K.R., Francis G.A. (2015). The Central Role of Proprotein Convertase Subtilisin/Kexin Type 9 in Septic Pathogen Lipid Transport and Clearance. Am. J. Respir. Crit. Care Med..

[14] Walley K.R., Thain K.R. (2014). PCSK9 is a critical regulator of the innate immune response and septic shock outcome. Sci. Transl. Med..

[15] Robinson J.G., Farnier M. (2015). Efficacy and safety of alirocumab in reducing lipids and cardiovascular events. N. Engl. J. Med..

[16] Karatasakis A., Danek B.A. (2017). Effect of PCSK9 Inhibitors on Clinical Outcomes in Patients with Hypercholesterolemia: A Meta-Analysis of 35 Randomized Controlled Trials. J. Am. Heart Assoc..

[17] Choi J., Khan A.M. (2017). Efficacy and safety of proprotein convertase subtilisin-kexin type 9 (PCSK9) inhibitors, alirocumab and evolocumab, a post-commercialization study. Lipids Health Dis..

[18] Navarese E.P., Podhajski P. (2023). PCSK9 Inhibition During the Inflammatory Stage of SARS-CoV-2 Infection. J. Am. Coll. Cardiol..

[19] Evans L., Rhodes A. (2021). Surviving Sepsis Campaign: International Guidelines for Management of Sepsis and Septic Shock. Crit. Care Med..

[20] Manniello M., Pisano M. (2016). Alirocumab (Praluent): First in the New Class of PCSK9 Inhibitors. Pharm. Ther..

